# Supplementary material for: Causal interplay between lactose intolerance and gut microbiota: a combined bidirectional Mendelian randomization and in vivo validation study
Source: Front Nutr. 2026 Jun 1;13:1803337. doi: 10.3389/fnut.2026.1803337 (PMC13265576; doi:10.3389/fnut.2026.1803337)
Supplement: Supplementary file 1 [file Data_Sheet_1.zip › supplementary materials/Forward/leave one out cross validation/ebi-a-GCST90027480.finngen_R12_E4_LACTONAS.pdf]

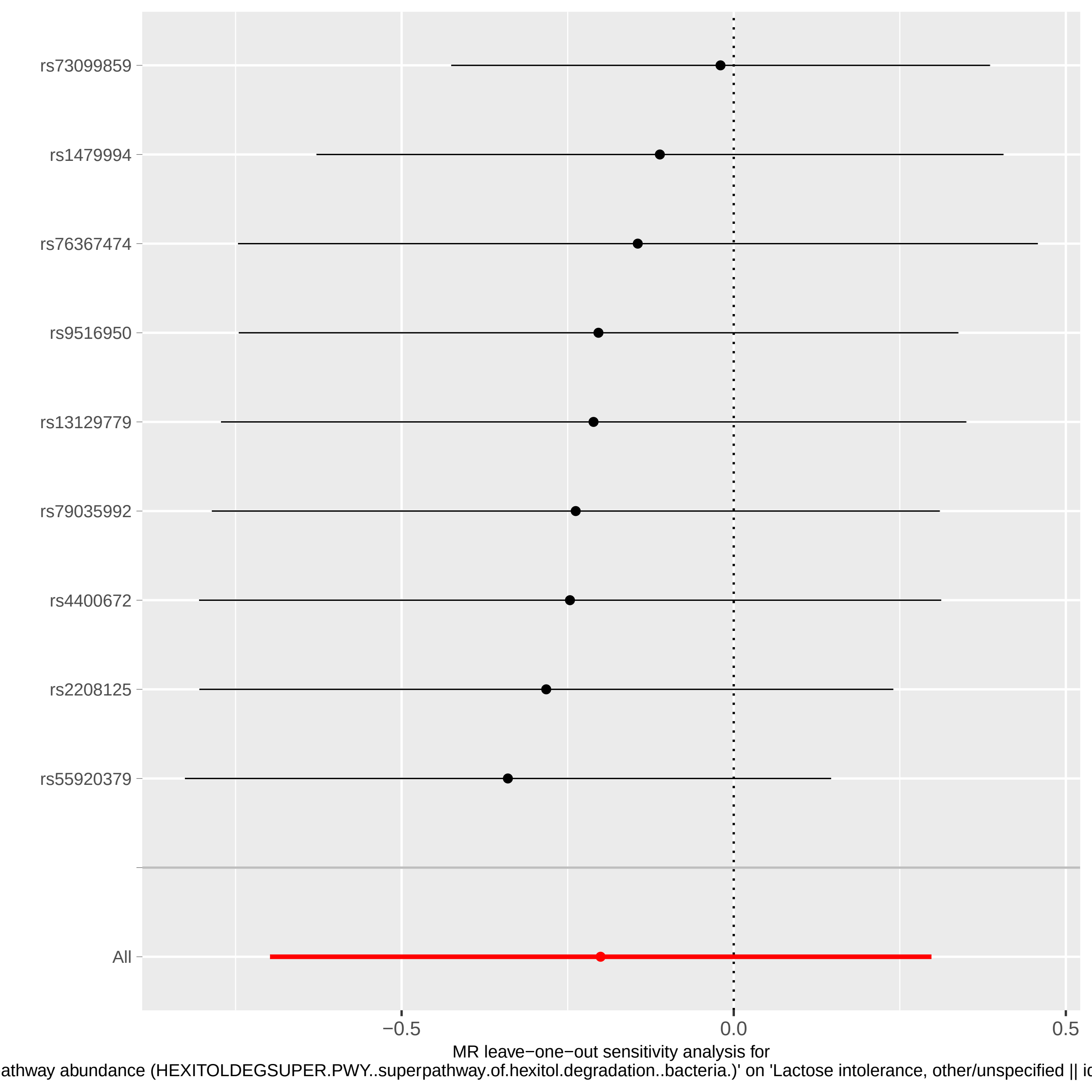

rs73099859
rs1479994
rs76367474
rs9516950
rs13129779
rs79035992
rs4400672
rs2208125
rs55920379
All
−0.5
0.0
0.5
MR leave−one−out sensitivity analysis for
athway abundance (HEXITOLDEGSUPER.PWY..superpathway.of.hexitol.degradation..bacteria.)' on 'Lactose intolerance, other/unspecified || ic
